# Modeling Impact and Cost-Effectiveness of Increased Efforts to Attract Voluntary Medical Male Circumcision Clients Ages 20–29 in Zimbabwe

**DOI:** 10.1371/journal.pone.0164144

**Published:** 2016-10-26

**Authors:** Katharine Kripke, Karin Hatzold, Owen Mugurungi, Gertrude Ncube, Sinokuthemba Xaba, Elizabeth Gold, Kim Seifert Ahanda, Natalie Kruse-Levy, Emmanuel Njeuhmeli

**Affiliations:** 1 Health Policy Project, Avenir Health, Washington, District of Columbia, United States of America; 2 Population Services International, Harare, Zimbabwe; 3 Zimbabwe Ministry of Health and Child Care, Harare, Zimbabwe; 4 Center for Communication Programs, Johns Hopkins Bloomberg School of Public Health, Baltimore, Maryland, United States of America; 5 The United States Agency for International Development (USAID), Washington, District of Columbia, United States of America; 6 USAID, Harare, Zimbabwe; Cardiff University, UNITED KINGDOM

## Abstract

**Background:**

Zimbabwe aims to increase circumcision coverage to 80% among 13- to 29-year-olds. However, implementation data suggest that high coverage among men ages 20 and older may not be achievable without efforts specifically targeted to these men, incurring additional costs per circumcision. Scale-up scenarios were created based on trends in implementation data in Zimbabwe, and the cost-effectiveness of increasing efforts to recruit clients ages 20–29 was examined.

**Methods:**

Zimbabwe voluntary medical male circumcision (VMMC) program data were used to project trends in male circumcision coverage by age into the future. The projection informed a base scenario in which, by 2018, the country achieves 80% circumcision coverage among males ages 10–19 and lower levels of coverage among men above age 20. The Zimbabwe DMPPT 2.0 model was used to project costs and impacts, assuming a US$109 VMMC unit cost in the base scenario and a 3% discount rate. Two other scenarios assumed that the program could increase coverage among clients ages 20–29 with a corresponding increase in unit cost for these age groups.

**Results:**

When circumcision coverage among men ages 20–29 is increased compared with a base scenario reflecting current implementation trends, fewer VMMCs are required to avert one infection. If more than 50% additional effort (reflected as multiplying the unit cost by >1.5) is required to double the increase in coverage among this age group compared with the base scenario, the cost per HIV infection averted is higher than in the base scenario.

**Conclusions:**

Although increased investment in recruiting VMMC clients ages 20–29 may lead to greater overall impact if recruitment efforts are successful, it may also lead to lower cost-effectiveness, depending on the cost of increasing recruitment. Programs should measure the relationship between increased effort and increased ability to attract this age group.

## Introduction

### History of VMMC implementation and strategy in Zimbabwe

In 2011, the World Health Organization (WHO) and the Joint United Nations Programme on HIV and AIDS (UNAIDS) released the “Joint Strategic Action Framework to Accelerate the Scale-Up of Voluntary Medical Male Circumcision for HIV Prevention in Eastern and Southern Africa 2012–2016” [1]. This document set a target of scaling up male circumcision (MC) to cover 80% of males ages 15–49 by 2016 in 14 countries with generalized HIV epidemics and low prevalence of male circumcision. Following the global guidance from WHO and UNAIDS, Zimbabwe conducted a series of epidemiological modeling exercises to estimate the impact of scaling up voluntary medical male circumcision (VMMC) on transmission of HIV in Zimbabwe. Analyses conducted in 2008 estimated that the greatest reduction in incidence for the medium term would come from circumcising men ages 20–29, and that in the long term, circumcising infants or adolescent boys (younger than 19 years of age) would result in the largest impact [2,3]. In response to this and additional costing and modeling conducted in 2010 [4], the Zimbabwe Ministry of Health set a target to scale up VMMC to 1.3 million males ages 13–29 between 2008 and 2015 [5].

As of June 2015, the national VMMC program had circumcised 509,753 males, of whom 441,133 were ages 13–29, representing 34% of the national VMMC target of 1.3 million males in this age group. The circumcisions conducted by the end of 2014 in Zimbabwe were projected to avert 6,300 HIV infections by 2025, with coverage estimated at 19% among 10- to 29-year-olds and 18% among 15- to 29-year-olds [6].

### Age-specific analyses of VMMC in the fourteen priority countries

As implementers rolled out VMMC programs in Zimbabwe and the other VMMC priority countries in eastern and southern Africa, it became evident that recruiting adolescents ages 10–19 was much easier than recruiting adult men for VMMC, and that few men over the age of 35 were accessing VMMC services [7, 8]. In some communities where traditional circumcision is practiced, circumcision during adolescence is normative [9], whereas circumcision for older men can be seen as shameful [10]. In addition, barriers to circumcision that exist for older men, such as time away from work, are less of an issue for adolescents [11]. Partly as a result of these observations, our team developed the Decision Makers’ Program Planning Tool (DMPPT), Version 2.0 to examine the impact and cost-effectiveness of circumcising different age groups of males [12].

The five DMPPT 2.0 country application papers in this collection [13–17] all compare hypothetical scenarios in which MC coverage is scaled up to 80% in various age groups. The overall conclusions of these papers, similar to the outcomes of modeling conducted previously for Zimbabwe [2,3], are that the greatest immediate impact (largest reduction of HIV incidence over five years) can be achieved by circumcising males ages 20–29 or 20–34, and that these age groups are important for efficiency (number of VMMCs per HIV infection averted) and cost-effectiveness (cost per HIV infection averted) of the program. Having reviewed these analyses, international donors are promoting increasing coverage among males ages 20–29, to increase the impact and cost-effectiveness of the program. However, the analyses upon which these policy recommendations were based assumed that it would be possible to achieve 80% coverage in each age group, and that the unit cost of VMMC (the cost per VMMC, including the direct cost of the circumcision and all associated program costs, including demand creation, facilities, management, quality assurance, etc.) would be the same regardless of client age. To the contrary, implementation experience suggests that reaching 80% coverage among men over age 20 will be difficult without increased demand creation focused on this age group, combined with potentially costly changes in program implementation, such as evening and weekend hours and other approaches to make VMMC services more adult-friendly. In addition, if the efforts to increase numbers of clients in this age group are not as successful as hoped, services may be underutilized, leading to even higher costs per circumcision [18]. The effectiveness of these program innovations is untested. There are no data to show whether or not increasing investment in attracting adult men to VMMC will succeed, and if it does, to what extent. Thus, international donors’ identification of males ages 20–29 as the age group having the greatest and most cost-effective impact on HIV prevention may require refinement based on implementation realities.

### Exploring cost-effectiveness of increasing coverage among men ages 20–29

Zimbabwe VMMC program trends suggest that if the VMMC program in Zimbabwe continues without any specific efforts to attract older men, coverage will likely plateau at a lower level for the men ages 20 and above. We used data on coverage trends by age for the national VMMC program in Zimbabwe to make predictions about the level at which coverage would plateau in the different age groups: the base scenario for our analyses. We projected the impact and cost-effectiveness of this scenario, which we considered more realistic than previous explorations that assume it is possible to reach 80% coverage in any age group without any additional investment in attracting the older men to services. We then assessed the primary research question in this paper: How would expending greater effort (and therefore funds) to increase coverage among clients ages 20–29 affect the impact and cost-effectiveness of the VMMC program? Because no data about the relationship between cost and increasing coverage by age group are available, we created modeling scenarios to explore this question based on arbitrary assumptions about how increased effort may lead to increased coverage. We contrast these more realistic scenarios with one that assumes that reaching 80% coverage among all males ages 10–29 can be achieved with the same VMMC unit cost across all age groups (similar to the analyses conducted in the other countries).

## Methods

### DMPPT 2.0 model

The DMPPT 2.0 model is described in detail in Kripke, Opuni, Schnure, et al. [12]. Briefly, DMPPT 2.0 is a simple compartmental model, implemented in Microsoft Excel 2010, that is designed to analyze the effects of age at circumcision on program impact and cost. The DMPPT 2.0 model tracks the number of circumcised males in newborns and in each five-year age group over time, taking into account age progression and mortality. The model calculates discounted VMMC program costs and HIV infections averted in the population each year in a user-specified VMMC scale-up strategy, compared with a counterfactual scenario in which the MC prevalence remains unchanged. The MC prevalence prior to initiation of the VMMC program (“baseline MC prevalence”) is assumed to be due to traditional or other circumcisions that continue to be conducted at the same rate after VMMC program initiation.

### Zimbabwe data sources

The DMPPT 2.0 model is populated with population, mortality, and HIV incidence and prevalence projections from an external source. For the Zimbabwe country application, we used the national Spectrum/AIM model [19], which projects population size, mortality, and HIV prevalence and incidence based on data empirically collected from the country. Population by age and year, mortality by age and year, annual number of male births, and HIV incidence by age and year were exported from this Spectrum/AIM file into a national Zimbabwe DMPPT 2.0 file.

Numbers of VMMCs conducted in the country each year through December 2014, disaggregated by client age, were provided by the Zimbabwe Ministry of Health and Child Care (MOHCC) on October 18, 2015 (S1 Table). The MC prevalence by age group in the model base year (2014) was derived from the Zimbabwe Demographic and Health Survey 2010–2011 [20]. The unit cost of VMMC used in the analysis started at US$109 (all subsequent references to currency are in U.S. dollars), based on Zimbabwe MOHCC data [5].

### Impact of scaling up MC coverage to 80% in each age group

To estimate the impact of scaling up MC to 80% coverage in separate five-year age groups, the model scaled up MC coverage within each individual five-year age group from the baseline level in 2014 to the target in 2018 while holding coverage constant at the baseline MC prevalence for the other age groups. The scale-up applied a linear interpolation between the baseline MC prevalence for each age group in 2014 and the 80% target coverage in 2018. After 2018, the coverage for each age group was maintained at 80%. For more details on the methods behind the results in this paper surrounding modeled incidence reduction from providing VMMC to individual age groups, and modeled impact of scaling up VMMC to progressively lower age groups, see Kripke, Chen, and Vazzano, et al. [15].

### Calculation of MC coverage from VMMC program data

The remainder of this paper is based on scenarios derived by projecting historical trends in MC coverage by age group and year from Zimbabwe’s VMMC program. Total MC coverage in year *t* and age group *a* (*C*_,*a*,*t*_) is given by the baseline MC prevalence for age group *a* plus the MC coverage resulting from the VMMC program for age group *a*:

$$C_{a,t}=C_{baseline,a,t}+C_{VMMC,a,t}$$

Male circumcision coverage resulting from the VMMC program was calculated from the age-disaggregated VMMC program statistics presented in S1 Table as follows:

$$C_{VMMC,a,t}=\frac{M_{VMMC,a,t}}{P_{a,t}}$$

where (*C*_*VMMC*,*a*,*t*_) is the MC coverage in age group *a* in year *t* resulting from the VMMC program, (*M*_*VMMC*,*a*,*t*_) is the number of males in age group *a* in year *t* who at some point in time had been circumcised through the VMMC program, and (*P*_*a*,*t*_) is the number of males in the population in age group *a* in year *t*.

*M*_*VMMC*,*a*,*t*_ is calculated as follows:

$$M_{VMMC,a,t}=M_{VMMC,a,t-1}*(1-\mu_{a,t-1})+(NewM_{VMMC,a,t-1}+AgingIn_{a,t-1}-AgingOut_{a,t-1})*\left(1-\frac{\mu_{a,t-1}}{2}\right)$$

The number of circumcised males in age group *a* at the beginning of year *t* was the number at the beginning of the previous year *t-1*, plus new VMMCs performed during year *t-1* and net VMMCs aging into and out of group *a*, all adjusted for mortality.

The increase in MC coverage in year *t* (*I*_*t*_) is given by:

$$I_{t}=C_{a,t}-C_{a,t-1}$$

### Derivation of scenarios from trends in coverage resulting from the VMMC program

To assess the impact, cost, and cost-effectiveness of increasing MC coverage among males ages 10–29 above what historical program trends would indicate, we used four scenarios. The first scenario assumed scale-up to 80% coverage among males ages 10–29, with the same unit cost ($109) for each five-year age group. Scale-up was linear from baseline levels in 2014 to 80% in 2018 and was maintained at 80% thereafter.

The base scenario was created as follows: For each age group (10–14, 15–19, 20–24, 25–29, and 30–49), we plotted the annual increase in MC coverage, calculated from the VMMC program data as described above, for each year from 2011 to 2014. We plotted the annual *increase* in MC coverage rather than the annual MC coverage, because we observed that the VMMC program was expanding exponentially each year, and we wished to project the level at which MC coverage would plateau in each age group if the program continued to expand at the same rate in each age group until it reached saturation, which we defined as 80% MC coverage among males ages 10–19.

**Fig 1:**
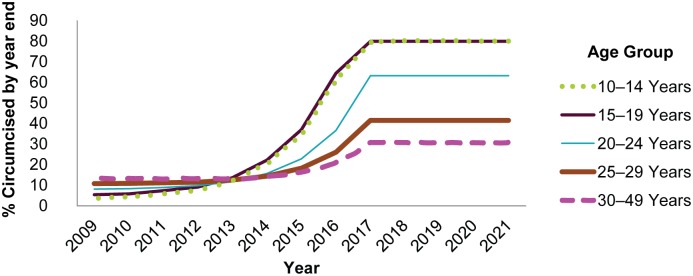
MC historical coverage through 2014 and target coverage after 2014 in base scenario.

An exponential trend line was fit in Microsoft Excel to the data for each age group (S1 Fig). S2 Table shows the formulas and R^2^ values for each trend line. In the base scenario, the increases in MC coverage for 2015–2018 for each age group were projected based on the indicated equation, with maximum possible MC coverage set to 80%. Based on this projection, the 10–14 and 15–19 year age groups reached 80% coverage by the end of 2017. We assumed that once the program reached 80% coverage of 10- to 19-year-olds, it would no longer be possible to increase MC coverage in the older age groups without additional demand creation and program modifications specifically targeted to these age groups. Therefore, in the base scenario, target coverage in the model from 2018 on was maintained constant for all age groups based on the coverage achieved by the end of 2017 (Fig 1). In the DMPPT 2.0 model, if the actual MC coverage in any given year is higher than the target coverage, no circumcisions are done in that age group. Therefore, after 2017, modeled circumcisions continued in the 10–14 year age group to maintain the 80% coverage, and they ceased among males ages 15 and older. Increases in coverage among males ages 15 and older after 2017 occurred as a result of circumcised males aging in from the younger age groups, but these are not shown in Fig 1, which shows the target, not actual coverage after 2014.

Scenarios A and B were created as follows: The coverage targets for the 10–14 and 15–19 year age groups were the same as in the base scenario. The annual increases in coverage in the 20–24 year age group were twice that of the base scenario for the years 2015–2016. Accordingly, the unit cost for the 20–24 year age group was double that used in the base scenario, because we made the arbitrary assumption that doubling the unit cost would double the increase in MC coverage in each year, up to a maximum total coverage of 80%. Coverage for the 20–24 year age group plateaued at 80% by the end of 2017. The annual increases in coverage in the 25–29 year age group, as well as the unit cost for this age group, were double that of the base scenario for Scenario A and triple that of the base scenario for Scenario B for the years 2015–2016. Coverage for the 25–29 year age group plateaued at 80% by the end of 2017 for Scenario B. Table 1 summarizes these four scenarios.

**Table 1:** Target Coverage by 2018 for Three Scenarios.

		Age Groups (Years of Age)				
Scenario	Description	10–14	15–19	20–24	25–29	30–49
	2014% MC coverage	21	22	16	14	14
80% Scenario	Unit cost	$109	$109	$109	$109	$109
	Increase % MC coverage 2014–2018	59	58	64	66	17
	2018% MC coverage target	80	80	80	80	31
Base Scenario	Unit cost	$109	$109	$109	$109	$109
	Increase % MC coverage 2014–2018	59	58	48	27	17
	2018% MC coverage target	80	80	63	42	31
Scenario A	Unit cost	$109	$109	$218	$218	$109
	2x 20–29 increase % MC coverage 2014–2018	59	58	64	54	17
	2018% MC coverage target	80	80	80	69	31
Scenario B	Unit cost	$109	$109	$218	$327	$109
	2x 20–24; 3x 25–29 increase % MC coverage 2014–2018	59	58	64	66	17
	2018% MC coverage target	80	80	80	80	31

The following model outputs for each scenario were measured over the 15-year period 2015–2029, inclusive: the total number of HIV infections averted in the population (including secondary HIV infections averted among females); the total cost of the VMMC program; the percentage of HIV infections averted; the number of VMMCs per HIV infection averted; and the cost per HIV infection averted. Costs, numbers of circumcisions, and infections averted were all discounted at a rate of 3% per year.

## Results

### Impact of reaching 80% MC coverage in each age group

**Fig 2:**
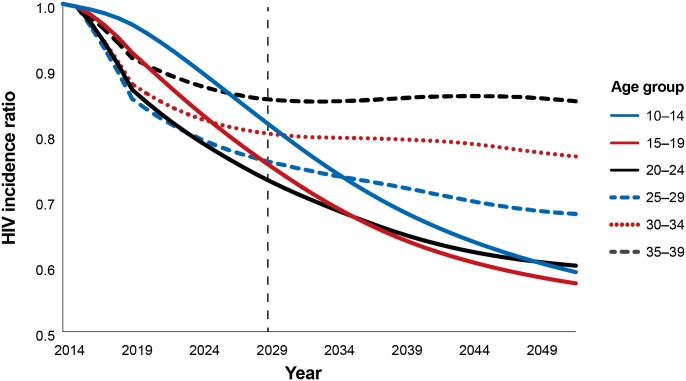
Reduction in HIV incidence with provision of VMMC to males, by age group, 2014–2050. The HIV incidence ratio represents the incidence in the scale-up scenario divided by the incidence in a population where circumcision is not scaled up over baseline levels. HIV incidence is in the entire population—males and females. Each line represents the HIV incidence ratio under a scenario in which only the indicated five-year age group is circumcised. In each age group, MC is scaled up to 80% coverage 2014–2018 and maintained at that level from 2018 forward. The dashed vertical line represents a 15-year period from the base year.

Prior analyses in other countries [12] projected the impact and cost-effectiveness of scaling up to 80% MC coverage in various age groups. Fig 2 shows a series of hypothetical scenarios in which VMMC is scaled up to 80% coverage in individual five-year age groups in Zimbabwe by 2018 and maintained at that level of coverage thereafter. Each line in Fig 2 represents a hypothetical scenario in which males only of the indicated age group are circumcised; those circumcised males age over time in the model, and reductions in incidence across the entire population are calculated. Assuming it is possible to reach 80% coverage in each age group, circumcising men ages 15–19, 20–24, and 25–29 would provide the greatest reduction in HIV incidence over the next 15 years (2015–2029). Circumcising clients in the younger age groups (ages 10–14, 15–19, and 20–24) would provide the greatest benefit over the long term under these assumptions.

### Age distribution of VMMC clients in Zimbabwe through 2014

**Fig 3:**
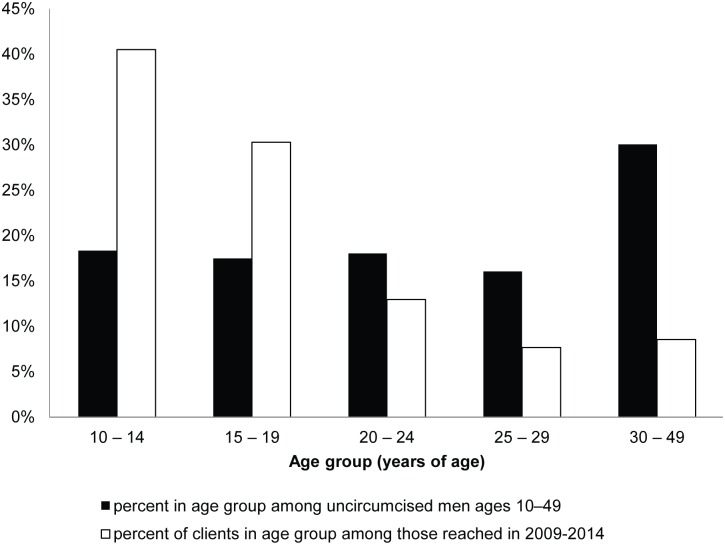
Age distribution of VMMC clients compared with the age distribution of potential clients. VMMC clients are shown in white bars; the age distribution of uncircumcised males in the overall population of 10- to 49-year-old males (potential clients) is shown in black bars.

The age distribution of VMMC clients in Zimbabwe has been biased toward adolescents. Clients ages 10–19 have been coming in at a higher rate and clients ages 20 and older have been coming in at a lower rate than would be expected based on the age distribution of uncircumcised men (Fig 3). Although the program aimed to circumcise males ages 13–29 according to the national strategy, 70% of VMMC clients in the Zimbabwe VMMC program through 2014 were between the ages of 10 and 19. As of the end of 2014, Zimbabwe had reached an estimated 21% MC coverage (including both traditional circumcisions and VMMCs) among 10- to 14-year-olds, 22% coverage among 15- to 19-year-olds, 16% coverage among 20- to 24-year-olds, and 14% coverage among 25- to 29-year-olds. These coverage levels represented increases of 17%, 17%, 8%, and 4% in coverage over baseline levels for these four respective age groups [6]. If these trends continue, the VMMC program will reach 80% coverage among adolescents ages 10–19 long before reaching this level of coverage among adults ages 20–29.

**Fig 4:**
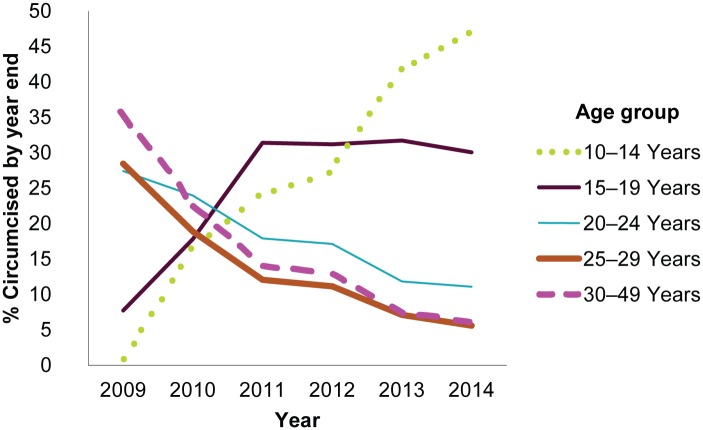
Trends in the proportion of each age group among VMMC clients over time.

Furthermore, the relative proportion of clients ages 10–14 has been increasing over time, whereas the proportion of clients over age 20 has been decreasing. The proportion of clients ages 15–19 appears to have stabilized in the past four years (Fig 4).

### MC coverage scenarios based on program trends through 2014

Given trends in the distribution of VMMCs by age group, we projected that implementers would not reach 80% MC coverage among the age groups above age 19 without significant additional effort and cost to recruit adults to access VMMC services. We asked at what level of MC coverage each age group would plateau if current trends continue. We assumed that as services continued to expand throughout the country, demand would saturate in each age group either when MC coverage reached 80% in a given age group or when MC coverage reached 80% in the 10–19 year age group, whichever came first (Fig 1). The assumption behind this was that this pattern would be followed within each VMMC catchment area as services were rolled out across the country, and this assumption became the base scenario for the analyses.

We then hypothesized that it would be possible to increase coverage of the 20–29 year age group, by putting increased effort and resources into interventions tailored to this age group. In the absence of costing data by age segment, we made an arbitrary assumption that a linear relationship exists between the unit cost for clients ages 20–29 (a proxy for effort) and the percentage of the target population ages 20–29 circumcised each year, such that doubling the VMMC unit cost would double the percentage of the target population circumcised, and so on. Thus we created two additional scenarios, as Table 1 shows in detail.

**Fig 5:**
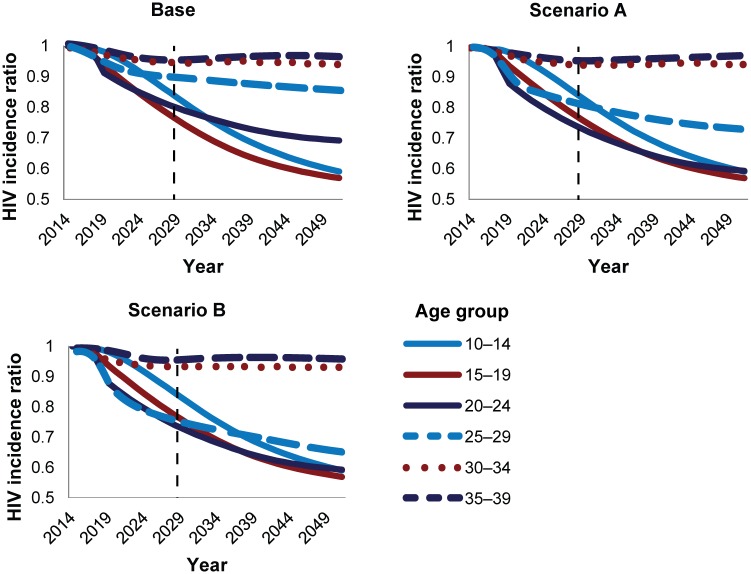
Modeled impact of providing VMMC to individual age groups in the various scenarios.

Fig 5 shows the reduction in HIV incidence that can be achieved by scaling up to the scenario targets from Table 1 in each individual age group by 2018 (as with Fig 2, each line represents a hypothetical scenario in which only males of that age group are circumcised). Unlike in Fig 2, in which MC coverage was scaled up to 80% in each age group by 2018, in the base scenario, the greatest reduction in HIV incidence after 15 years (by 2029) is achieved by circumcising clients ages 15–19 and 20–24. As coverage increases among males ages 20–24 from the base scenario to Scenario A, the impact of circumcising that age group increases, and it initially surpasses the impact of circumcising the 10–14 and 15–19 year age groups. When the program circumcises clients ages 25–29, the impact progressively increases as coverage is scaled up to 42%, 69%, and 80% coverage in the base, A, and B scenarios, respectively. Nonetheless, the impact of circumcising males ages 25–29 plateaus at a lower level than it does with the younger age groups, even in Scenario B, where MC coverage reaches 80%.

While Fig 5 shows the reduction in HIV incidence that can be achieved by circumcising each individual five-year age group, this is not the same as the contribution of each age group to the reduction in incidence when broader age bands are circumcised. Therefore, it is useful to assess the contribution of each age group when males across the entire 10–34 age range are circumcised, and the coverage plateaus for each age group at the level specified in each scenario.

**Fig 6:**
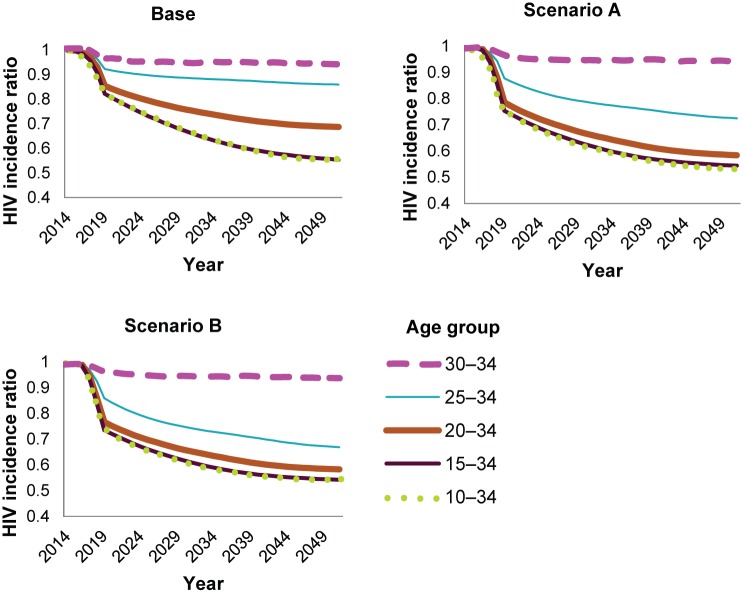
Impact of scaling up VMMC to progressively lower age groups in the various scenarios.

Fig 6 compares the impact of adding progressively lower age groups for each of the three scenarios, showing the relative contribution of each age group to the reduction in incidence in each scenario. In the base scenario, the greatest reduction in HIV incidence is contributed by including the 20–24 year age group, because the distance between the 20–34 and 25–34 lines is greatest. In Scenarios A and B, when the 25–29 year age group is scaled up to 69% and 80% coverage, respectively, this age group makes the greatest contribution to reducing HIV incidence, as evidenced by the fact that, for these scenarios, the distance between the 25–34 and 30–34 lines is the greatest. This demonstrates that the contribution of each age group to the impact on HIV incidence partially depends on the level of coverage that can be achieved in that age group. The male HIV incidence is highest in the 25–29 and 30–34 year age groups (S2 Fig). This is why even when coverage in the 25–29 year age group is less than that of the younger age groups (such as in Scenario A, when coverage in this age group is 69% and coverage among those ages 10–24 is 80%), the 20- to 29-year-olds can provide the greatest contribution to HIV incidence reduction. Inclusion of the 10–14 year age group provides a negligible additional HIV incidence reduction, as the majority of these clients are not sexually active and would therefore benefit just as much by being circumcised between ages 15 and 19.

Table 2 shows the cost and impact of the various scenarios. The base scenario has the lowest total cost and lowest number of HIV infections averted. The 80% scenario has the lowest numbers of VMMCs per HIV infection averted and the lowest cost per HIV infection averted. In comparison with the base scenario, Scenarios A and B have progressively higher total cost, total HIV infections averted, and cost per HIV infection averted and progressively lower numbers of VMMCs per HIV infection averted.

**Table 2:** Cost and Impact of the Four Scenarios, 2015–2029.

	80% Scenario	Base Scenario	Scenario A	Scenario B
HIV Infections Averted	87,000	63,000	78,000	83,000
Number of VMMCs	3,850,000	3,490,000	3,850,000	3,940,000
Total Cost	$420,000,000	$380,000,000	$516,000,000	$593,000,000
% HIV Infections Averted	48	35	44	46
# VMMCs per HIV Infection Averted	45	55	49	48
Cost per HIV Infection Averted	$4,800	$6,000	$6,600	$7,200

No data are currently available about the relationship between cost and increased coverage for a specific age group, so the unit cost assumptions used in Scenarios A and B in Table 2 are arbitrary. Therefore we conducted a sensitivity analysis in which the unit cost in Scenario A was multiplied by a series of factors compared with the unit cost of the base scenario, and assessed how that would impact the total cost and cost per HIV infection averted. (All other parameters of Scenario A remained the same, so the other outputs shown in Table 2 did not change in the sensitivity analysis.) Table 3 shows the results of this sensitivity analysis.

**Table 3:** Sensitivity Analysis of Unit Cost for Circumcising Males Ages 20–29 in Scenario A^1^.

Unit Cost Multiplier for Ages 20–29	Unit Cost for Ages 20–29	Total Cost	Cost per HIV Infection Averted
1	$109	$420,000,000	$5,400
1.1	$120	$429,000,000	$5,500
1.2	$131	$439,000,000	$5,600
1.3	$142	$449,000,000	$5,700
1.4	$153	$458,000,000	$5,800
1.5	$163	$468,000,000	$6,000
1.6	$174	$478,000,000	$6,100
1.7	$185	$487,000,000	$6,200
1.8	$196	$497,000,000	$6,300
1.9	$207	$507,000,000	$6,500
2	$218	$516,000,000	$6,600

^1^The unit cost of circumcising men ages 20–29 in the base scenario ($109) was multiplied by the indicated unit cost multiplier for these age groups in Scenario A. All other parameters for Scenario A remained the same as those shown in Table 1.

If no additional cost per VMMC is required to increase coverage among males ages 20–29 (unit cost multiplier 1; unit cost $109), the total cost of Scenario A is $420 million—the same as for the 80% scenario, but with fewer HIV infections averted, because Scenario A does not reach 80% coverage among males ages 25–29. (The reason the total cost is the same is that the rate of scale-up for the 80% scenario is linear, while that of Scenario A is exponential, and the two scenarios just happen to end up with roughly the same total number of VMMCs throughout the scale-up phase.) The cost per HIV infection averted for Scenario A in this case is $5,400, which is higher than that of the 80% scenario but lower than that of the base scenario. When the cost per VMMC for males ages 20–29 is 50% higher than in the base scenario (unit cost multiplier 1.5; unit cost $163), the cost per HIV infection averted in Scenario A, $6,000, is the same as in the base scenario, and the total cost of Scenario A, $468 million, is higher than that of the 80% scenario. If the unit cost required to increase coverage among males ages 20–29 is more than 50% higher than that of the base scenario, the cost per HIV infection averted is higher than that of the base scenario.

### Limitations

This study has several limitations. The general limitations of the DMPPT 2.0 model are discussed in the methods paper in this collection [12] and they also apply to this analysis. The primary limitations of this particular analysis are the assumptions about the level at which male circumcision coverage would plateau in each age group, and the fact that the actual relationship between cost and increased coverage by age group is unknown. Pilot programs to recruit greater numbers of clients over the age of 20 are under way not only in Zimbabwe (as described) but also in South Africa and Tanzania. It will be crucial for these programs to collect data about the relationship between program cost and increased recruitment among this age group.

## Discussion

In this paper, we predicted that VMMC coverage in the age groups above age 19 would plateau at a lower level than in the younger age groups, based on historical trend data from the VMMC program and knowledge of the sociocultural factors that make VMMC more desirable to adolescents. If our prediction turns out to be the case, the relative contribution of each age group to HIV incidence reduction will be based on a combination of the level of coverage at which the program plateaus for each age group and the HIV incidence in that age group and the next higher age group. In the base scenario explored in this paper, the greatest contribution to HIV incidence reduction comes from circumcising males between the ages of 15 and 19. Increasing coverage among males ages 20–24 and 25–29 increases the contribution of these age groups to HIV incidence reduction.

No data have been published or are otherwise available showing how much it costs to increase coverage among males ages 20 and above. We hypothesized that it would be possible to increase coverage of the 20–29 year age group by putting increased effort and resources into demand creation interventions tailored to this age group, such as outreach to specific locations where older men (20–29) convene. In addition, increased uptake of services among this older age group would likely entail structural changes at service delivery sites to make them more attractive to the older males, such as “VIP services,” offering extended hours, and separate transport and waiting areas for adults, who are often embarrassed to mix with adolescents. Additional formative research could be conducted to tailor campaigns to the factors that motivate the older males in their specific contexts, including socioemotional factors that may move them to action.

In the absence of actual data about the cost required to increase coverage among 20- to 29-year-olds, we conducted an analysis using an arbitrary assumption that a linear relationship exists between the age-specific unit cost and the percentage of the target population ages 20–29 circumcised in each year, for the sake of exploring what might happen. Given this assumption, our analysis demonstrated that increased coverage among these age groups would lead to higher costs per HIV infection averted (lower cost-effectiveness) of the VMMC program than did scenarios that assumed the cost to reach men ages 20–29 would be the same as the cost to reach adolescents ages 10–14. In Scenarios A and B, in which increased resources were used to increase coverage among the population ages 20–29 compared to the base scenario, the number and percentage of HIV infections averted, total cost, and cost per HIV infection averted were progressively higher than in the base scenario, while the numbers of VMMCs per HIV infection averted were progressively lower.

A sensitivity analysis in which the unit cost for circumcising males ages 20–29 was varied for Scenario A showed that the cost per HIV infection averted was equal in the base scenario and Scenario A when the unit cost of circumcising males ages 20–29 in Scenario A was 1.5 times that of the base scenario. Below this threshold, the cost per HIV infection averted was lower in Scenario A than in the base scenario, and vice versa. Therefore, if additional resources are available to increase coverage among men ages 20–29, it might be possible to increase the overall impact of the VMMC program, and doing so could also make the program either more or less cost-effective. The actual relationship between cost and increased recruitment of clients in the 20- to 29-year age group will determine whether these efforts make the program more cost-effective or less cost-effective, so collecting these data is crucial.

Zimbabwe is trying to increase representation of men ages 20–29 among VMMC clients. In order to scale up demand and encourage men, especially those 20–29 years old, to get circumcised, the country began a comprehensive research program in 2015 to map a man’s journey to circumcision and identify points at which interventions could be strategically placed to increase demand for circumcision. Based on this research, the Government and implementing partners developed a detailed communications and marketing strategy targeting specific segments of men. In 2016, the country began implementing that strategy, accompanied by costing studies. The data collected can shed insights on the feasibility of this strategy and inform further analyses about cost-effectiveness of increasing demand among 20- to 29-year-old men.

To date, seven countries (Malawi, South Africa, Swaziland, Tanzania, Uganda, Zambia, and Zimbabwe) have completed detailed country DMPPT 2 model applications to reexamine their age-targeting strategies for VMMC [13–17,21,22]. In most cases, these modeling exercises have led to revised age-specific targets in the countries’ VMMC operational plans.

The modeling presented here demonstrates that cost-effectiveness analyses of age targeting by VMMC programs need to take two related factors into account that were not considered previously: (1) the feasibility of reaching high levels of coverage among specified target age groups, and (2) if reaching these high levels of coverage is possible at all, how much that will cost, in comparison with the costs of programs as currently implemented. Zimbabwe is modifying its VMMC program to attract more males ages 20–29. Even if the country’s age-specific recruitment efforts are successful, increasing the program’s overall impact on HIV incidence, the added investment required may also lead to lower cost-effectiveness. Data collected from this program in Zimbabwe and similar efforts in other countries can be applied to cost-effectiveness analyses to inform future age-specific VMMC strategies in Zimbabwe and in other VMMC priority countries.

## Supporting Information

S1 Table Number of VMMCs Conducted in Zimbabwe, Disaggregated by Age Group and Year. Note: Data were obtained from national program records. Because VMMCs for ages 30–49 were not available disaggregated by five-year age groups, they were disaggregated based on the age distribution of circumcisions conducted in Malawi in PEPFAR FY 2013, based on PEPFAR program data. VMMCs for ages 50+ were put into the 50–54 age group in the DMPPT 2.0 model.
(XLSX)

S2 Table Equations and R^2^ Values for Trends Fit to MC Coverage Increases for 2011–2014. The x-values in the equation represent the year minus 2010 (the first year of the curve projection).
(DOCX)

S1 Fig Annual increase in male circumcision coverage, by age group. Each solid line presents data from a specific VMMC client age group. Male circumcision (MC) coverage is measured at the beginning of each year. Trend lines (dotted lines) were generated from Microsoft Excel 2013 using an exponential fit.
(TIF)

S2 Fig Age-specific male HIV incidence in 2014, from Zimbabwe Spectrum/AIM Model. (TIF)
