# Correction: Modeling Impact and Cost-Effectiveness of Increased Efforts to Attract Voluntary Medical Male Circumcision Clients Ages 20–29 in Zimbabwe

**DOI:** 10.1371/journal.pone.0169696

**Published:** 2017-01-03

**Authors:** Katharine Kripke, Karin Hatzold, Owen Mugurungi, Gertrude Ncube, Sinokuthemba Xaba, Elizabeth Gold, Kim Seifert Ahanda, Natalie Kruse-Levy, Emmanuel Njeuhmeli

There is an error in the Funding statement. The number for the Cooperative Agreement Project SOAR (Supporting Operational AIDS Research) is incorrect. The correct number is OAA-A-14-00060.
