# Supplementary material for: Modeling Impact and Cost-Effectiveness of Increased Efforts to Attract Voluntary Medical Male Circumcision Clients Ages 20–29 in Zimbabwe
Source: PLoS One. 2016 Oct 26;11(10):e0164144. doi: 10.1371/journal.pone.0164144 (PMC5082672; doi:10.1371/journal.pone.0164144)
Supplement: S2 Table — The x-values in the equation represent the year minus 2010 (the first year of the curve projection). (DOCX) [file pone.0164144.s002.docx]

S2 Table. Equations and R^2^ Values for Trends Fit to MC Coverage Increases for 2011–2014. The x-values in the equation represent the year minus 2010 (the first year of the curve projection).

| Age Group | Exponential Fit | R^2^ |
| --- | --- | --- |
| 10–14 years | y=.0073e^0.5892x^ | 0.8646 |
| 15–19 years | y=0.0069e^0.6136x^ | 0.9339 |
| 20–24 years | y=0.0027e^0.6557x^ | 0.9671 |
| 25–29 years | y=0.0012e^0.6942x^ | 0.9656 |
| 30–49 years | y=0.0004e^0.7891x^ | 0.9773 |
