# Supplementary figures and images for: Modeling Impact and Cost-Effectiveness of Increased Efforts to Attract Voluntary Medical Male Circumcision Clients Ages 20–29 in Zimbabwe
Source: PLoS One. 2016 Oct 26;11(10):e0164144. doi: 10.1371/journal.pone.0164144 (PMC5082672; doi:10.1371/journal.pone.0164144)

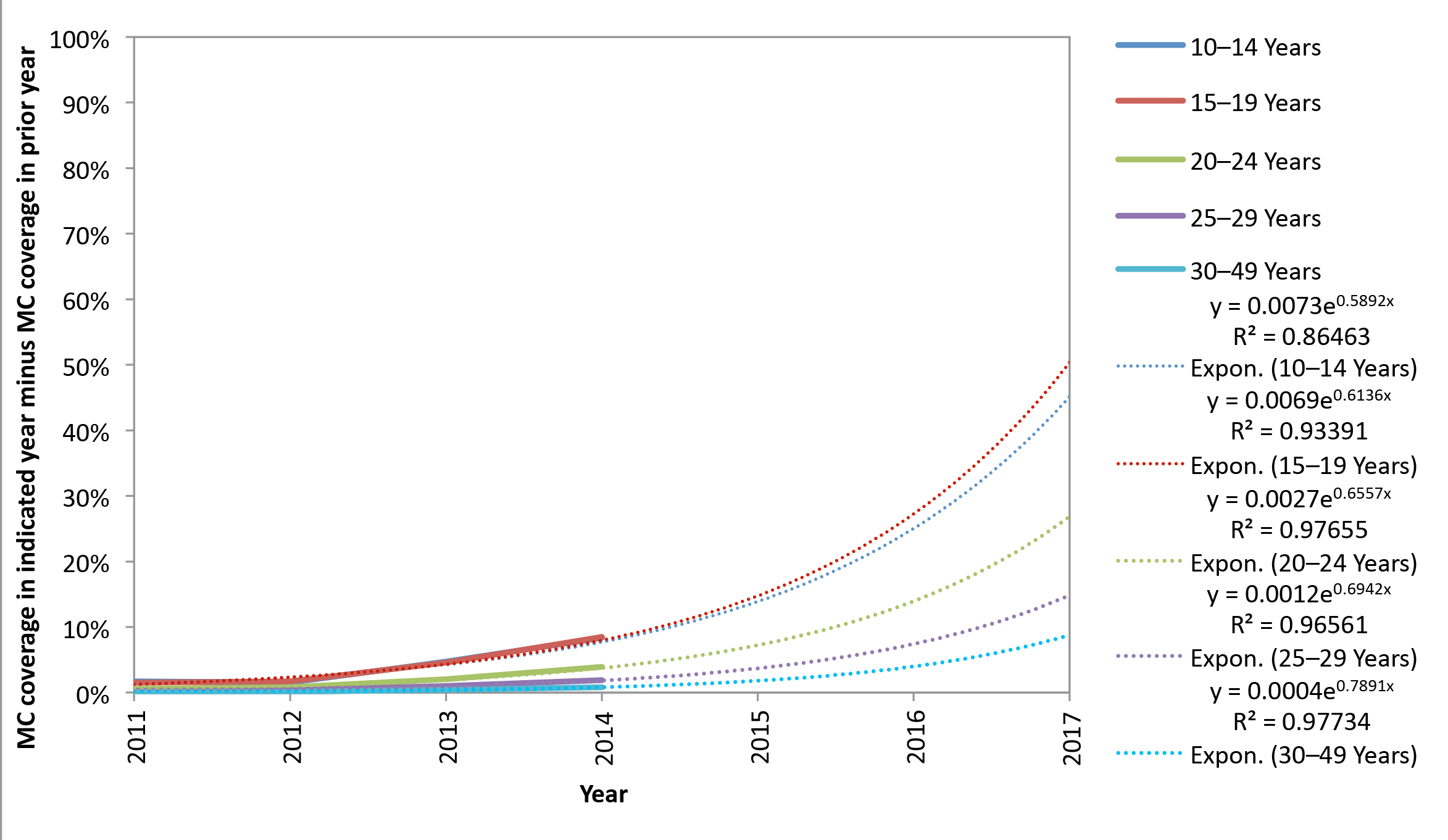

Supplement: S1 Fig — Each solid line presents data from a specific VMMC client age group. Male circumcision (MC) coverage is measured at the beginning of each year. Trend lines (dotted lines) were generated from Microsoft Excel 2013 using an exponential fit. (TIF) [file pone.0164144.s003.tif]

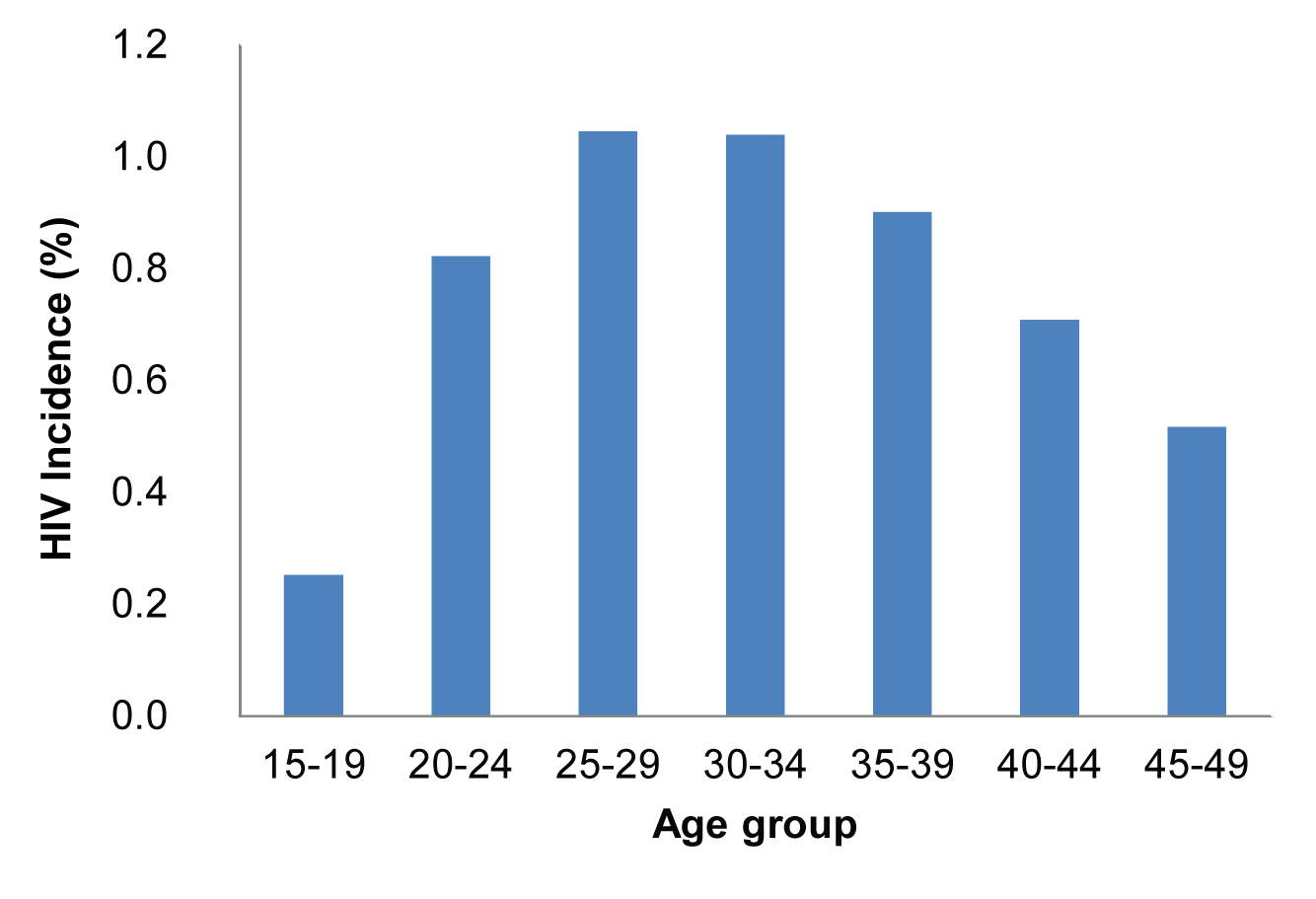

Supplement: S2 Fig — (TIF) [file pone.0164144.s004.tif]
